# Haploinsufficiency of *BCL11A* associated with cerebellar abnormalities in 2p15p16.1 deletion syndrome

**DOI:** 10.1002/mgg3.289

**Published:** 2017-05-22

**Authors:** Hiroko Shimbo, Takayuki Yokoi, Noriko Aida, Seiji Mizuno, Hiroshi Suzumura, Junichi Nagai, Kazumi Ida, Yumi Enomoto, Chihiro Hatano, Kenji Kurosawa

**Affiliations:** ^1^ Clinical Research Institute Kanagawa Children's Medical Center Yokohama Japan; ^2^ Division of Medical Genetics Kanagawa Children's Medical Center Yokohama Japan; ^3^ Division of Radiology Kanagawa Children's Medical Center Yokohama Japan; ^4^ Department of Pediatrics Aichi Human Service Center Central Hospital Kasugai Japan; ^5^ Department of Pediatrics Dokkyo Medical University School of Medicine Tochigi Japan; ^6^ Laboratory Medicine Kanagawa Children's Medical Center Yokohama Japan

**Keywords:** *BCL11A*, cerebellar abnormalities, chromosome 2p15p16.1 deletion syndrome, chromosome 2p16.1 deletion, intellectual disability, neurodevelopmental delay, structural brain abnormality.

## Abstract

**Background:**

Chromosome 2p15p16.1 deletion syndrome is a rare genetic disorder characterized by intellectual disability (ID), neurodevelopmental delay, language delay, growth retardation, microcephaly, structural brain abnormalities, and dysmorphic features. More than 30 patients with 2p15p16.1 microdeletion syndrome have been reported in the literature.

**Methods:**

Molecular analysis was performed using microarray‐based comparative genomic hybridization (array CGH). Clinical characteristics and brain magnetic resonance imaging features of these patients were also reviewed.

**Results:**

We identified four patients with ID, neurodevelopmental delay, brain malformations, and dysmorphic features; two patients with 2p15p16.1 deletions (3.24 Mb, 5.04 Mb), one patient with 2p16.1 deletion (1.12 Mb), and one patient with 2p14p16.1 deletion (5.12 Mb). Three patients with 2p15p16.1 deletions or 2p16.1 deletions encompassing *BCL11A*,*PAPOLG*, and *REL* showed hypoplasia of the pons and cerebellum. The patient with 2p14p16.1 deletion, which did not include three genes showed normal size and shape of the cerebellar hemispheres and pons.

**Conclusion:**

The zinc finger transcription factor *BCL11A* associated with the BAF chromatin remodeling complex has been identified to be critical for neural development and *BCL11A* haploinsufficiency is closely related to cerebellar abnormalities.

## Introduction

Chromosome 2p15p16.1 microdeletion syndrome (OMIM: 612513) is characterized by intellectual disability (ID), neurodevelopmental delay, structural brain abnormalities, and dysmorphic features. Rajcan‐Separovic et al. (2007) first reported a 2p15p16.1 deletion syndrome and so far, more 30 patients have been reported to have overlapping deletions encompassing this region (Table 1) (Rajcan‐Separovic et al. 2007; Chabchoub et al. 2008; de Leeuw et al. 2008; Liang et al. 2009; Felix et al. 2010; Prontera et al. 2011; Hucthagowder et al. 2012; Piccione et al. 2012; Florisson et al. 2013; Hancarova et al. 2013; Fannemel et al. 2014; Jorgez et al. 2014; Peter et al. 2014; Balci et al. 2015; Basak et al. 2015; Ottolini et al. 2015; Ronzoni et al. 2015; Shimojima et al. 2015; Bagheri et al. 2016). A recent functional study by Bagheri et al. (2016) suggested that *BCL11A* (MIM 606557), *REL* (MIM 164910), and *XPO1* (MIM 602559) represented candidate genes for structural brain abnormalities in 2p15p16.1 microdeletion syndrome. One of these genes, Kruppel‐like transcription factor *BCL11A* (B cell CLL/lymphoma 11A, also known as *CTIP1*) associated with the BRG1/BRM‐associated factor (BAF) chromatin remodeling complex has been identified to be critical for neural development (Lessard et al. 2007; Kadoch et al. 2013). Balci et al. (2015) reported a patient with a 2p16.1 deletion including only *BCL11A* and Dias et al. (2016) recently described 11 patients with *BCL11A* heterozygous intragenic mutations (three missense and eight nonsense/frameshift). These patients had an ID with structural brain abnormalities. The common brain malformations in 2p15p16.1 deletion syndrome were observed hypoplasia of the corpus callosum and cortical dysplasia. Here, we report four patients with ID, neurodevelopmental delay, language delay, brain malformations, and dysmorphic features; two of these patients had 2p15p16.1 deletions, one had a 2p14p16.1 deletion, and one had a 2p16.1 deletion. Three of these patients with deletions including *BCL11A*,* PAPOLG* (MIM 616865), and *REL* demonstrated a small pons with proportional cerebellar vermis, along with hemispheric hypoplasia and a dilated caudal subarachnoid space of the posterior fossa. The patient with the 2p14p16.1 deletion not including *BCL11A*,* PAPOLG*, and *REL* was microcephalic but showed a normal pons and cerebellum. We summarize brain magnetic resonance imaging (MRI) analysis along with genetic and clinical data to define the associated phenotype spectrum.

**Table 1:**
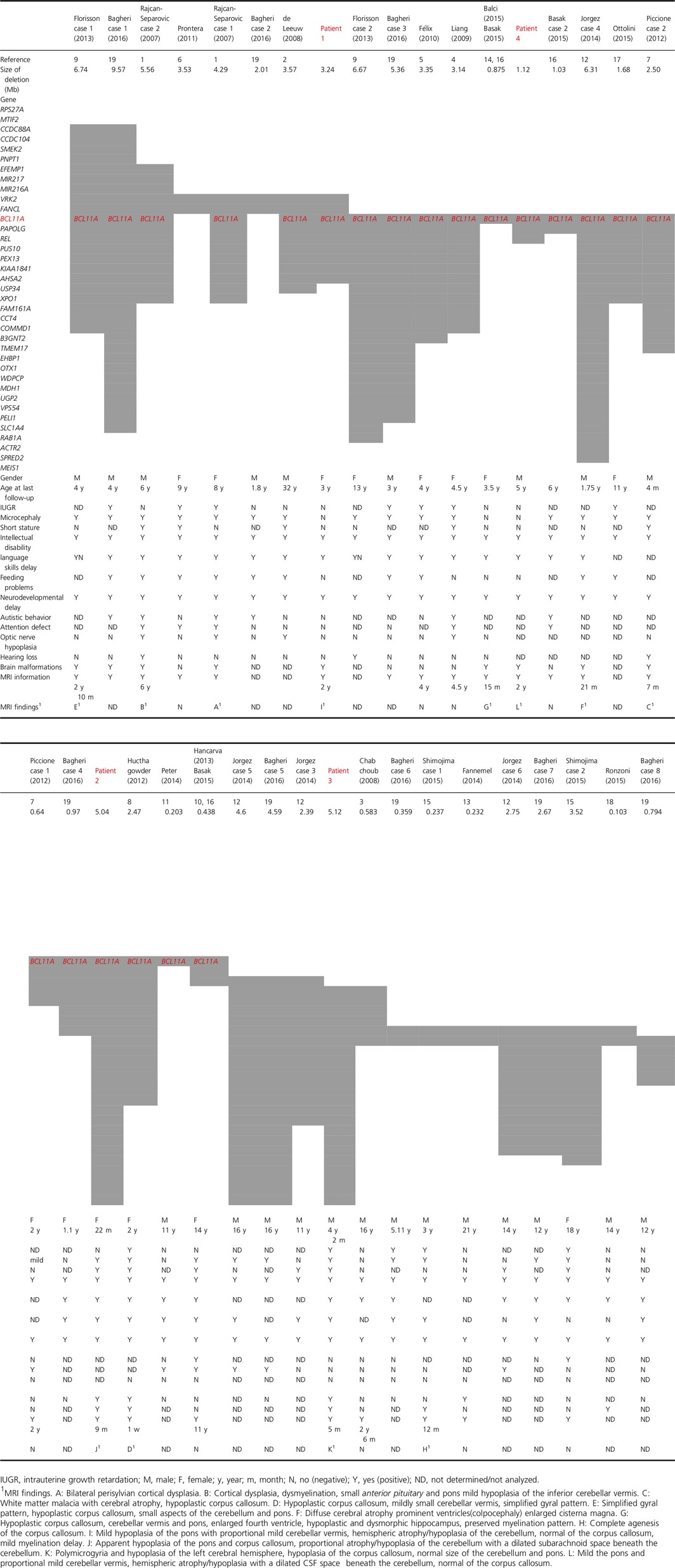
Comparison of clinical features for our four patients and 33 reported cases

## Clinical Reports

Clinical characteristics of four patients and 33 reported cases are summarized in Table 1.

### Patient 1

Our first patient was a 3‐year‐old girl who was born at 39 weeks' gestation with a birth weight of 3048 g (−0.1 SD) and a length of 50 cm (+0.6 SD). Her parents were healthy. She was able to lift her head at the age of 3 months, sit up independently at 10 months, walk by holding onto furniture at 22 months, and walk independently at 36 months. She began babbling at 12 months but did not speak any meaningful words. She was referred to our institution for developmental delay at the age of 18 months. Physical examination at 18 months revealed a height of 81.0 cm (−0.6 SD), a weight of 10.78 kg (−0.9 SD), and an occipitofrontal circumference (OFC) of 44.3 cm (−1.5 SD), indicating normal growth. She had strabismus, telecanthus, broad nasal bridge, retrognathia, and low‐set ears. MRI of her brain at 2 years of age revealed mild hypoplasia of the pons with proportional mild cerebellar vermis, hemispheric atrophy/hypoplasia of the cerebellum, normal of the corpus callosum, mild myelination delay.

### Patient 2

Our second patient was a 22‐month‐old girl who was born at 39 weeks' gestation with a birth weight of 2484 g (−1.5 SD), a length of 44.5 cm (−2.3 SD), and an OFC of 31 cm (−1.6 SD). Her parents were also healthy. She had several dysmorphic features including microcephaly and low anal atresia during the neonatal period. She also had severe neurodevelopmental delay. She was able to lift her head and roll over at the age of 10 months but was not able to sit up independently at 2 years of age. She was referred to our institution for identification of the malformation syndrome. Physical examination at 18 months, a height of 70.0 cm (−3.3 SD), a weight of 7.0 kg (−2.6 SD), and an OFC of 38 cm (−6.0 SD) were observed; these findings were indicative of growth retardation and microcephaly. She had strabismus, telecanthus, a broad nasal bridge, low‐set ears, and mitral regurgitation. MRI of her brain at 9 months of age revealed apparent hypoplasia of the pons and corpus callosum, proportional atrophy/hypoplasia of the cerebellum with a dilated subarachnoid space beneath the cerebellum.

### Patient 3

Our third patient was a 4 years and 2 months old boy who was born at 40 weeks' gestation with a birth weight of 2440 g (−1.9 SD), a length of 47.5 cm (−1.2 SD), and an OFC of 31.5 cm (−1.4 SD). His parents were healthy. He was referred to our institution for blepharophimosis at the age of 1 month. He responded to visual tracking at 4 months and laughed while being cradled at 7 months. He was able to roll over at 8 months and sit up independently at the age of 15 months. He was able to walk independently at 29 months. He had not spoken any meaningful words by 3 years of age. Physical examination at 4 years and 2 months revealed a height of 97 cm (−1.1 SD) and a weight of 11.6 kg (−2.0 SD). He had a broad forehead and displayed conditions such as blepharophimosis, hypertelorism, downslanted palpebral fissure, ptosis, epicanthus inversus, small mouth, micrognathia, cleft palate, hypotonia, small penis, and long toes. MRI of his brain at 5 months of age revealed polymicrogyria and hypoplasia of the left cerebral hemisphere, hypoplasia of the corpus callosum, normal size of the cerebellum and pons.

### Patient 4

Our fourth patient was a 5‐year‐old boy who was born at 40 weeks' gestation with a birth weight of 3286 g (+0.2 SD), a length of 50 cm (+0.2 SD), and an OFC of 33.5 cm (±0 SD). Both his parents and two elder sisters were healthy. He was able to lift his head at 4 months, sit up independently at 10 months, walk holding onto furniture at 17 months, and walk independently at 30 months. He had spoken only one word by the age of 3 years and 6 months. He was referred to our institution for developmental delay at 4 years and 6 months. Physical examination revealed that his height was 105 cm (−0.3 SD), his weight was 16.15 kg (−0.6 SD), and his OFC was 49.5 cm (−0.6 SD), indicating normal growth. He had strabismus, ocular hypertelorism, short palpebral fissures, broad nasal bridge, arched eyebrows, low‐set ears, and a transverse palmar crease on both hands. MRI of his brain at 2 years of age revealed mild the pons and proportional mild cerebellar vermis, hemispheric atrophy/hypoplasia with a dilated CSF space beneath the cerebellum, normal of the corpus callosum.

### MRI findings

**Figure 1:**
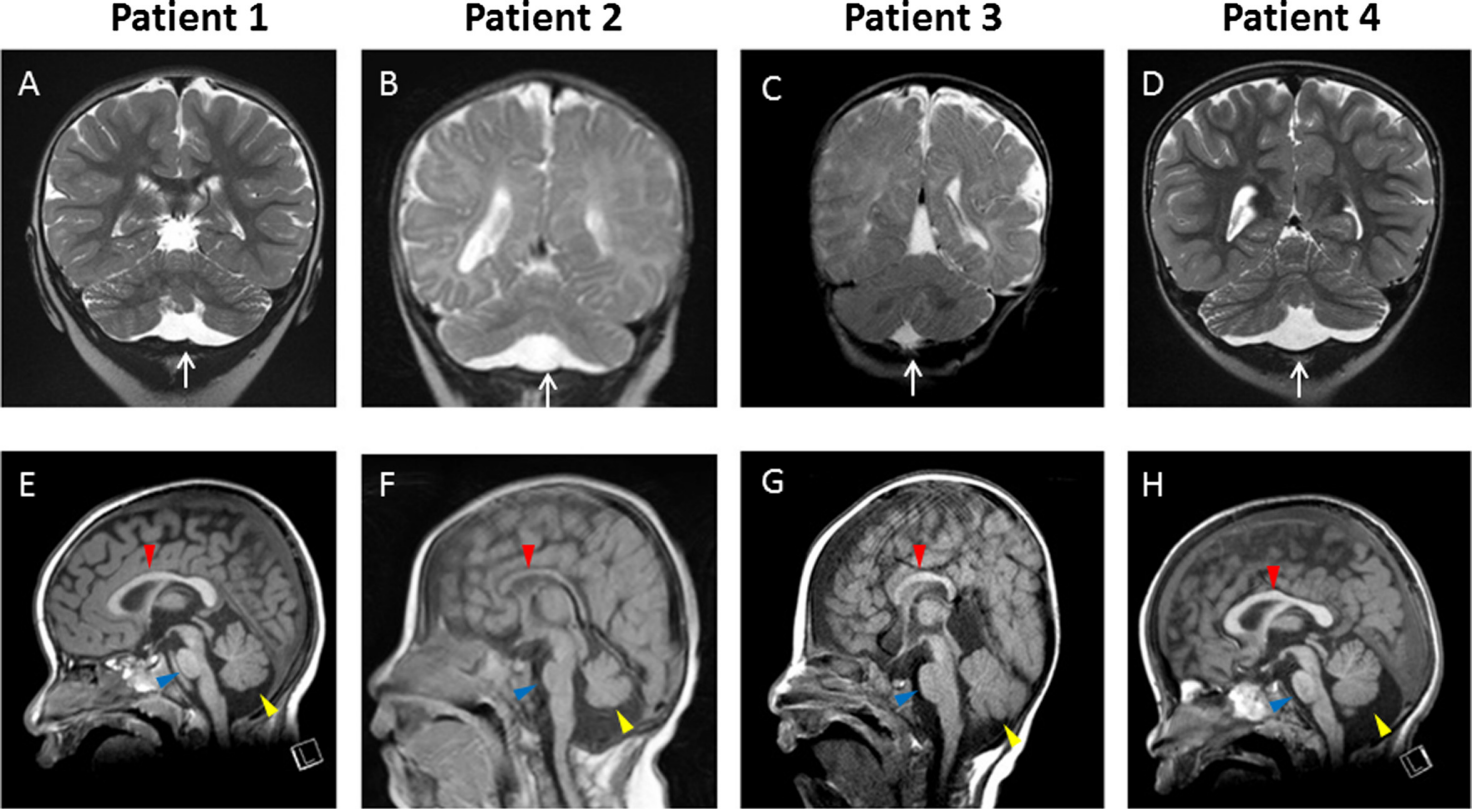
Selected coronal and sagittal MR images in four patients. (A and E) Patient 1: A 3‐year‐old female with 2p15p16.1 deletion (3.24 Mb). The cranial MRI was performed at 2 years of age. (A) T2‐weighted coronal and (E) T1‐weighted sagittal MR images show (A) mild hypoplastic, flattened cerebellar hemispheres with proportionally reduced size of the vermis and dilated subarachnoid space beneath the cerebellum (white arrow), and (E) mild hypoplasia of the pons (blue arrowhead), a slightly small cerebellar vermis (yellow arrowhead), and intact corpus callosum (red arrowhead). (B and F) Patient 2: A 22‐month‐old female with 2p15p16.1 deletion (5.04 Mb). The cranial MRI was performed at 9 months of age. (B) T2‐weighted coronal and (F) T1‐weighted sagittal MR images show (B) hypoplastic, flattened cerebellar hemispheres with proportionally reduced size of the vermis with dilated subarachnoid space beneath the cerebellum (white arrow), and (F) prominent hypoplasia of the pons (blue arrowhead), very small cerebellar vermis (yellow arrowhead), and thin corpus callosum (red arrowhead). (C and G) Patient 3: A 4 years 2 months old male with 2p14p16.1 deletion (5.12 Mb). The cranial MRI was performed at 5 months of age. (C) T2‐weighted coronal and (G) T1‐weighted sagittal MR images show (G) normal size and shape of the cerebellar hemispheres, cerebellar vermis (yellow arrowhead), pons (blue arrowhead), and small corpus callosum (red arrowhead), and (C) without dilated subarachnoid space beneath the cerebellum (white arrow). (D and H) Patient 4: A 5‐year‐old male with 2p16.1 deletion (1.12 Mb). The cranial MRI was performed at 2 years of age. (D) T2‐weighted coronal and (H) T1‐weighted sagittal MR images show (D) mild hypoplastic, flattened cerebellar hemispheres with proportionally reduced size of the vermis with dilated subarachnoid space beneath the cerebellum (white arrow), and (H) mild hypoplasia of the pons (blue arrowhead), small cerebellar vermis (yellow arrowhead), and intact corpus callosum (red arrowhead).

We present the MRI findings in Figure 1.

## Materials and Methods

### Ethical compliance

Signed informed consent was obtained from the patient's parents in accordance with the Kanagawa Children's Medical Center Review Board and Ethics Committee.

### Array CGH and FISH analyses

The UCSC database (genome build human genome 19 [hg19]) was used to map genomic coordinates and identify genes within regions where copy number had been altered (please refer to the Supporting Information).

## Results

**Figure 2:**
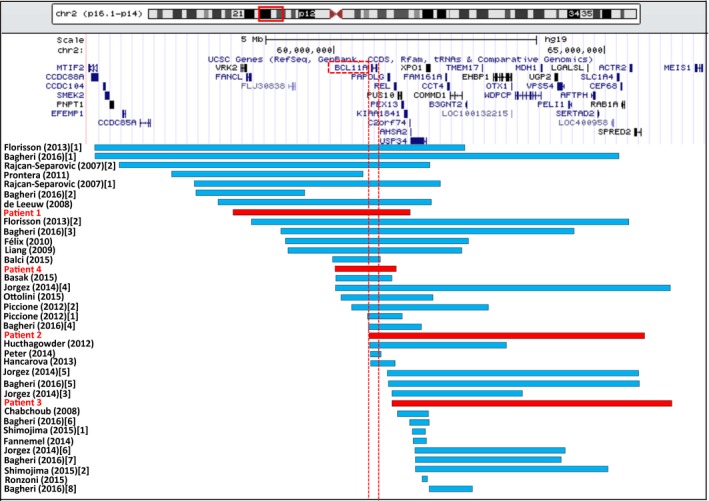
A schematic representation of the 2p15.16.1 microdeletions in four patients in this study and previous 33 cases. The blue bars are reported cases and the red bars present our 4 new cases. Vertical dashed lines indicate the beginning and end of the *BCL11A*.

Genomic and clinical findings of four new cases and 33 previously reported cases with 2p15p16.1 deletion syndrome are summarized in Table 1. Common features are ID, neurodevelopmental delay, language delay, growth retardation, microcephaly, structural brain abnormalities, and dysmorphic features. Using the Agilent SurePrint G3 Human CGH Microarray, deletion size was confirmed from 1.12 to 5.12 Mb in our four patients and compared to previous 33 cases (Fig. 2). Patient 1 and Patient 2 had deletions of 3.24 Mb (positions 58,029,768–61,275,725; hg19) and 5.04 Mb (positions 60,676,037–65,731,798; hg19), respectively, in chromosome 2p15p16.1. In Patient 3, there was a 5.12‐Mb (positions 61,136,131–66,258,735; hg19) deletion in chromosome 2p14p16.1, and in Patient 4, we identified a 1.12 Mb deletion (positions 60,013,464–61,136,190; hg19), involving *BCL11A, PAPOLG*, and *REL* in 2p16.1 (Figure S1A–D). The deletion in the chromosomal region was also confirmed by a fluorescence in situ hybridization (FISH) analysis. These could not be detected in the parents, indicating that they were de novo deletions. Additional copy number variants (CNVs) of unknown clinical significance were identified in Patients 1, 2, and 3 (Supporting Information). There were no other significant CNVs in Patient 4.

## Discussion

In the present study, we report four new patients with overlapping deletions at the 2p15p16.1, 2p14p16.1, or 2p16.1. In previous reports, these deletions were associated with various clinical symptoms, including brain malformations, microcephaly, ID, delayed speech and language development, autistic behavior, and facial dysmorphisms (Rajcan‐Separovic et al. 2007; Chabchoub et al. 2008; de Leeuw et al. 2008; Liang et al. 2009; Felix et al. 2010; Prontera et al. 2011; Hucthagowder et al. 2012; Piccione et al. 2012; Florisson et al. 2013; Hancarova et al. 2013; Fannemel et al. 2014; Jorgez et al. 2014; Peter et al. 2014; Balci et al. 2015; Basak et al. 2015; Ottolini et al. 2015; Ronzoni et al. 2015; Shimojima et al. 2015; Bagheri et al. 2016). In this study, our two patients (Patients 1 and 2) exhibiting deletion at the 2p15p16.1 region and Patient 4 exhibiting deletion at the 2p16.1 region, involving *BCL11A*,* PAPOLG*, and *REL* had similar MRI findings, which showed proportional hypoplasia/atrophy of the cerebellar hemispheres along with vermis and hypoplastic pons in all three cases as well as intact corpus callosum in two cases (Fig. 1A, B, D, E, F, H). In contrast, MRI findings from Patient 3 exhibiting a deletion at the 2p14p16.1 region, which did not include *BCL11A*,* PAPOLG*, and *REL*, showed normal size and shape of the cerebellar hemispheres, cerebellar vermis, pons, and a small corpus callosum (Fig. 1C, G).

Bagheri et al. (2016) suggested the critical region of *BCL11A*,* REL*, and *XPO1* by gene knockdown of zebrafish orthologs in 2p15p16.1 microdeletion syndrome. Two of these genes, *BCL11A*,* REL* were included deletion region in Patients 1, 2, and 4. *BCL11A* is a strong candidate gene related to structural brain abnormalities on the basis of clinical reports of patients with large deletion, small deletion including only *BCL11A* (Balci et al. 2015), and intragenic mutation in *BCL11A* (Dias et al. 2016). Animal model studies also support the mechanism of brain development (Leid et al. 2004; John et al. 2012; Wiegreffe et al. 2015; Bagheri et al. 2016). *REL* is also candidate gene related to not only structural brain abnormalities but also abnormal growth and dysmorphism (Bagheri et al. 2016). However, patients with microdeletion including only *REL* or intragenic mutation in *REL* have not been reported in the literature.

Of 37 patients with 2p15p16.1, 2p14p16.1, or 2p16.1 deletion, 4 new cases and 33 previous cases are described (Table 1). Information on brain MRI is available for 23 patients and 13 of these patients were associated with structural brain abnormalities; microdeletion of chromosomal region encompassing the *BCL11A* gene was observed in 11/13 (three were our cases and eight previous cases) (Rajcan‐Separovic et al. 2007; Hucthagowder et al. 2012; Piccione et al. 2012; Florisson et al. 2013; Jorgez et al. 2014; Balci et al. 2015; Bagheri et al. 2016) and cerebellar abnormalities were seen in 6/11 including three our cases and three previously reported (Rajcan‐Separovic et al. 2007; Florisson et al. 2013; Balci et al. 2015). The other 10 patients appeared normal on MRI, although microdeletion of chromosomal region encompassing *BCL11A* was observed in six of these patients (Liang et al. 2009; Felix et al. 2010; Piccione et al. 2012; Hancarova et al. 2013; Basak et al. 2015; Bagheri et al. 2016). The common brain malformations in 2p15p16.1 deletion syndrome were observed hypoplasia of the corpus callosum and cortical dysplasia. However, mutations in this microdeletion syndrome cause variable neurodevelopmental phenotypes, some patients did not reveal abnormalities on MRI. Balci et al. reported a patient with a 2p16.1 deletion including *BCL11A* only, brain imaging showed hypoplasia of the pons and cerebellar vermis, bowing and thinning of the corpus callosum, mild cerebral atrophy and dysmorphic amygdale and hippocampi. Sagittal T2 WI at the midline also showed an enlarged third and fourth ventricles, broad communication of the fourth ventricle with an enlarged retrocerebellar cistern through the foramen of magendie (Balci et al. 2015). Rajcan‐Separovic et al. (2007) reported two patients who had large deletions including *BCL11A*. Both cases had cortical dysplasia and one of them also had an enlarged fourth ventricles, mild hypoplasia of the inferior cerebellar vermis, and small anterior pituitary and pons. Florisson et al. (2013) described two patients with craniosynostosis and microcephaly with large deletions including *BCL11A*. MRI of one patient showed a simplified gyral pattern, hypoplastic corpus callosum, small aspects of the cerebellum and pons. On the other hand, Peter et al. (2014) reported a patient with microdeletion that included only *BCL11A*, who had a mild ID, hypotonia, gross motor dyspraxia, and a severe speech sound disorder, however, unfortunately there was no reported brain MRI finding. Hancarva et al. (2013) described a patient with a smaller deletion including *BCL11A*,* PAPOLG*, and *REL*, who had a delayed psychomotor development and language skills, but brain MRI did not appear abnormal.

Dias et al. (2016) recently described in 11 patients with *BCL11A* haploinsufficiency, who showed a syndromic form of ID. Information on brain MRI is available for six patients. Five of these patients showed structural brain abnormalities and two of them showed hypoplasia/atrophy of the cerebellar vermis.

By the way, during mouse development, *Bcl11a* was expressed the in the cerebral cortex, hippocampus, and cerebellum (Leid et al. 2004). Mice with ubiquitous loss of *Bcl11a* led to perinatal lethality and conditional knockout models showed reduction of cortical thickness and layer disorganization (John et al. 2012; Wiegreffe et al. 2015). Dias et al. (2016) described that *Bcl11a* haploinsufficiency leads to microcephaly in mice with heterozygous deletion of *Bcl11a* in accordance with the human phenotype.

Kuo et al. (2010) reported that Bcl11A protein was identified as a calcium/calmodulin‐dependent serine protein kinase (CASK)‐binding protein from a yeast two‐hybrid analysis and CASK interacted with Bcl11A and regulates outgrowth and branching of axons.

The phenotype of atrophy/hypoplasia of the cerebellum in patients with microdeletions encompassing *BCL11A* was reminiscent of neurological disease caused by *CASK* (MIM 300749) mutations (Najm et al. 2008; Takanashi et al. 2010; Moog et al. 2011). Furthermore, infratentorial neuroradiological findings in most cases with *CASK* mutations described by Moog et al. (2011) are almost identical to those of our three present cases with deletions encompassing *BCL11A*, although MRI coronal image rhombencephalon in our patients were much milder. These neuroradiological similarities in these two abnormalities are interesting because cerebellar hypoplasia/atrophy is not always accompanied by dilated caudal subarachnoid space of the posterior fossa. Cerebellar hypoplasia has not been reported in these mutant mouse models (John et al. 2012; Wiegreffe et al. 2015; Dias et al. 2016), but *Bcl11a* might be related to the developing cerebellum from gene expression patterns (Leid et al. 2004) and CASK interacting (Kuo et al. 2010). Furthermore, the zinc finger transcription factor *BCL11A* is also a member of BAF complex which has been implicated in the modification of chromatin structure and represented an important cause of developmental disorders (Lessard et al. 2007; Kadoch et al. 2013). Recent studies indicate that *BCL11A* regulates neurodevelopmental pathways associated with autism spectrum disorder (Iossifov et al. 2012; de Rubeis et al. 2014). Further investigations have shown that cerebellum anomalies are strongly associated with autism spectrum and language disorders (Hodge et al. 2010). Therefore, *BCL11A* haploinsufficiency is closely related to abnormal cerebellum development and our present study supports the underlying mechanism of cerebellar hypoplasia in 2p15p16.1 deletion syndrome is consistent with the idea that Balci et al. (2015) previously suggested.

## Conflict of Interest

The authors have no conflicts of interest to disclose.

## Supporting information


**Figure S1.** Identification of the chromosomal deletions by analysis of Agilent SurePrint G3 60K array. (A) Patient 1 (2p15p16.1 deletion, 3.24 Mb from 58,029,768 to 61,275,725, hg19), (B) Patient 2 (2p15p16.1 deletion, 5.04 Mb from 60,676,037 to 65,731,798, hg19), (C) Patient 3 (2p14p16.1 deletion, 5.12 Mb from 61,136,131 to 66,258,735, hg19), and (D) Patient 4 (2p16.1 deletion, 1.12 Mb from 60,013,464 to 61,136,190, hg19). Blue (patient sample) and red (control sample) dots represent the log2 intensity ratios of the single nucleotide polymorphism probe. Arrows indicate loss of heterozygosity of the deletion.

**Appendix S1.** Array CGH, FISH and *BCL11A* copy number analyses.
