# Supplementary figures and images for: Haploinsufficiency of BCL11A associated with cerebellar abnormalities in 2p15p16.1 deletion syndrome
Source: Mol Genet Genomic Med. 2017 May 22;5(4):429–37. doi: 10.1002/mgg3.289 (PMC5511803; doi:10.1002/mgg3.289)

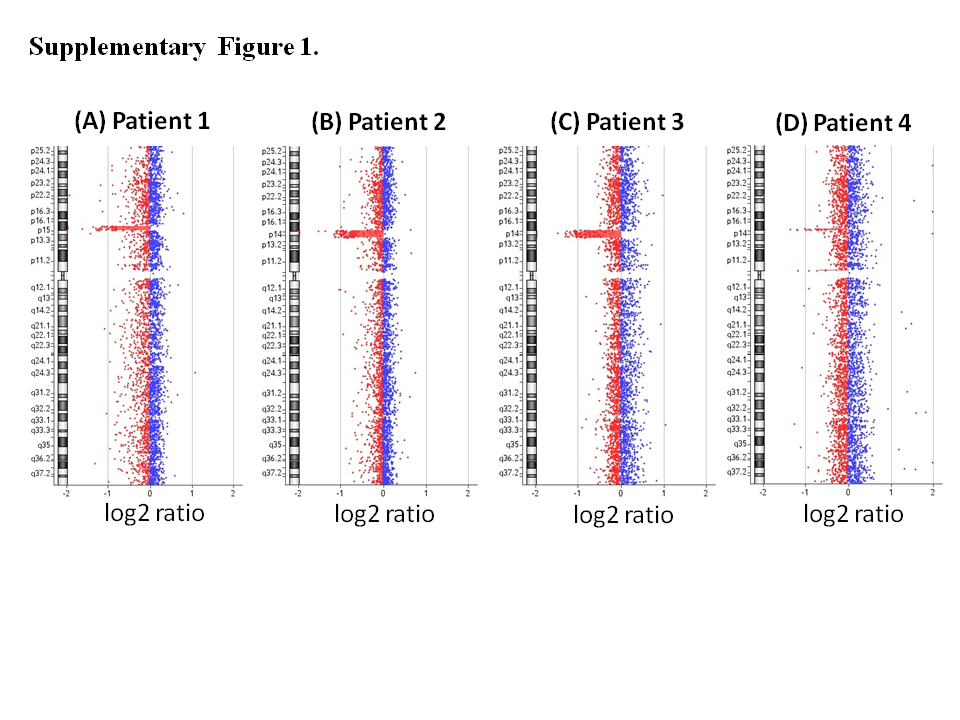

Supplement: Supplementary file 1 — Figure S1. Identification of the chromosomal deletions by analysis of Agilent SurePrint G3 60K array. (A) Patient 1 (2p15p16.1 deletion, 3.24 Mb from 58,029,768 to 61,275,725, hg19), (B) Patient 2 (2p15p16.1 deletion, 5.04 Mb from 60,676,037 to 65,731,798, hg19), (C) Patient 3 (2p14p16.1 deletion, 5.12 Mb from 61,136,131 to 66,258,735, hg19), and (D) Patient 4 (2p16.1 deletion, 1.12 Mb from 60,013,464 to 61,136,190, hg19). Blue (patient sample) and red (control sample) dots represent the log2 intensity ratios of the single nucleotide polymorphism probe. Arrows indicate loss of heterozygosity of the deletion. [file MGG3-5-429-s001.tif]
