# Supplementary material for: Haploinsufficiency of BCL11A associated with cerebellar abnormalities in 2p15p16.1 deletion syndrome
Source: Mol Genet Genomic Med. 2017 May 22;5(4):429–37. doi: 10.1002/mgg3.289 (PMC5511803; doi:10.1002/mgg3.289)
Supplement: Supplementary file 2 — Appendix S1. Array CGH, FISH and BCL11A copy number analyses. [file MGG3-5-429-s002.doc]

**Supplementary text**

Haploinsufficiency of *BCL11A* associated with cerebellar abnormalities in 2p15p16.1 deletion syndrome

Hiroko Shimbo, Takayuki Yokoi, Noriko Aida, Seiji Mizuno, Hiroshi Suzumura, Jun-ichi Nagai, Kazumi Ida, Yumi Enomoto, Chihiro Hatano, Kenji Kurosawa

**Abbreviations**

Array CGH, microarray-based comparative genomic hybridization; FISH, fluorescence in situ hybridization; qPCR, quantitative real-time PCR

**Methods**

**Array CGH and FISH analyses**

Genomic DNA was extracted from lymphocytes using Qiagen DNeasy Blood Extraction Kit (Qiagen, Gaithersburg, MD, USA). Array CGH was then performed in a clinical laboratory using the Agilent SurePrint G3 Human CGH Microarray Kit 8 × 60K (Agilent Technologies) according to the manufacture’s protocol. The UCSC database (genome build hg19) was used to map genomic coordinates and identify genes within regions where copy number had been altered. Data were analyzed using Agilent Genomic Workbench software (Agilent Technologies). The deletions were confirmed using custom-made probes for FISH [1].

**Results**

**Additional reported genomic findings**

Additional copy number variants (CNVs) of unknown clinical significance were identified at 8p11.22 dup (0.13Mb), 15q11.1-q11.2 del (2.0Mb) in patient 1, 14q11.2 (122kb), 15q11.1-q11.2 del (2.0Mb), Xq11-1-11.2 del (145kb) in patient 2, and 11p13 dup (195kb), 8p11.22 dup (0.13Mb), 10q21.3 del (266kb), 15q11.1-11.2 del (1.93Mb) in patient 3.

Reference

[1] Ishikawa A, Enomoto K, Tominaga M et al. Pure duplication of 19p13.3. Am J Med Genet A 2013: 161A (9): 2300-2304.
